# An unusual case report of indolent T-cell lymphoproliferative disorder with aberrant CD20 expression involving the gastrointestinal tract and bone marrow

**DOI:** 10.1186/s13000-018-0762-4

**Published:** 2018-10-20

**Authors:** Xingen Wang, Chi-Sing Ng, Cuimin Chen, Guangyin Yu, Weihua Yin

**Affiliations:** 1 Department of Pathology, Shenzhen Hospital of Peking University, Lianhua road 1120, Futian district of Shenzhen, Shenzhen, 518036 China; 2 Department of pathology, St. Teresa’s Hospital, Ma Tau Wai, Hong Kong

**Keywords:** Indolent T-cell lymphoproliferative disorder, Gastrointestinal tract, Aberrant CD20 expression

## Abstract

**Background:**

Indolent T-cell proliferative disorder of the GIT is a rare and provisional entity in the revised WHO 2016 classification. The patients usually have prolonged survival with persistent disease even without any treatment.

**Case presentation:**

The 46 years old male patient has been followed up for more than 6 years without chemotherapy. Repeated gastrointestinal biopsies showed expansion of the lamina propria extending to the submucosa by small to medium sized lymphocytes with minimal cytologic atypia. The lymphoid cells were positive for CD3, CD43, TIA-1, CD2, CD7 and the B-cell marker CD20; but negative for CD4, CD8, PAX5, CD56, cyclinD1, granzyme (GraB) and Epstein Barr virus-encoded RNA (EBER). Ki-67(MIB1) index was less than 10%. Molecular tests demonstrated a clonal rearrangement for T-cell receptor γ (TCR γ) gene but immunoglobulin chain (IgH, IgK, IgL) gene remained germline. Recognition of possible aberrant CD20 expression in indolent T-cell LPD is important to avoid potential diagnostic pitfall and improper treatment.

**Conclusions:**

We present an unusual case of indolent T-cell lymphoproliferative disorder with aberrant CD20 expression, Recognition of this unusual immunophenotype of indolent T-cell LPD of GI helps to eschew misdiagnosis of B-cell and other high grade lymphomas and inappropriate aggressive treatment.

## Background

About 20–25% of primary extranodal lymphomas occur in the GIT [1]. Intestinal T-cell lymphomas are less common than B-cell lymphomas and most are highly aggressive diseases [2] with frequent recurrence despite intensive multimodal therapy. The five-year survival rate is less than 20% [3]. Indolent T-cell proliferative disorder of the GIT is a rare and provisional entity in the revised WHO 2016 classification. The patients usually have prolonged survival with persistent disease even without any treatment [4].

We report an unusual case of indolent T-cell proliferative disorders of the GIT with aberrant expression of CD20. The lesion involved the stomach, ileum, colon and bone marrow without evidence of large cell transformation. The disease has not shown progression for about 6 years from the initial biopsies to the latest follow up without chemotherapy. To the best of our knowledge, this is the first report of aberrant CD20 expression in indolent T-cell LPD of the GIT. Recognition of this immunophenotypic aberrance is important to differentiate indolent T-cell LPD from small B-cell lymphoma of the GIT and to avoid mismanagement.

## Case presentation

### Clinical history

**Fig. 1:**
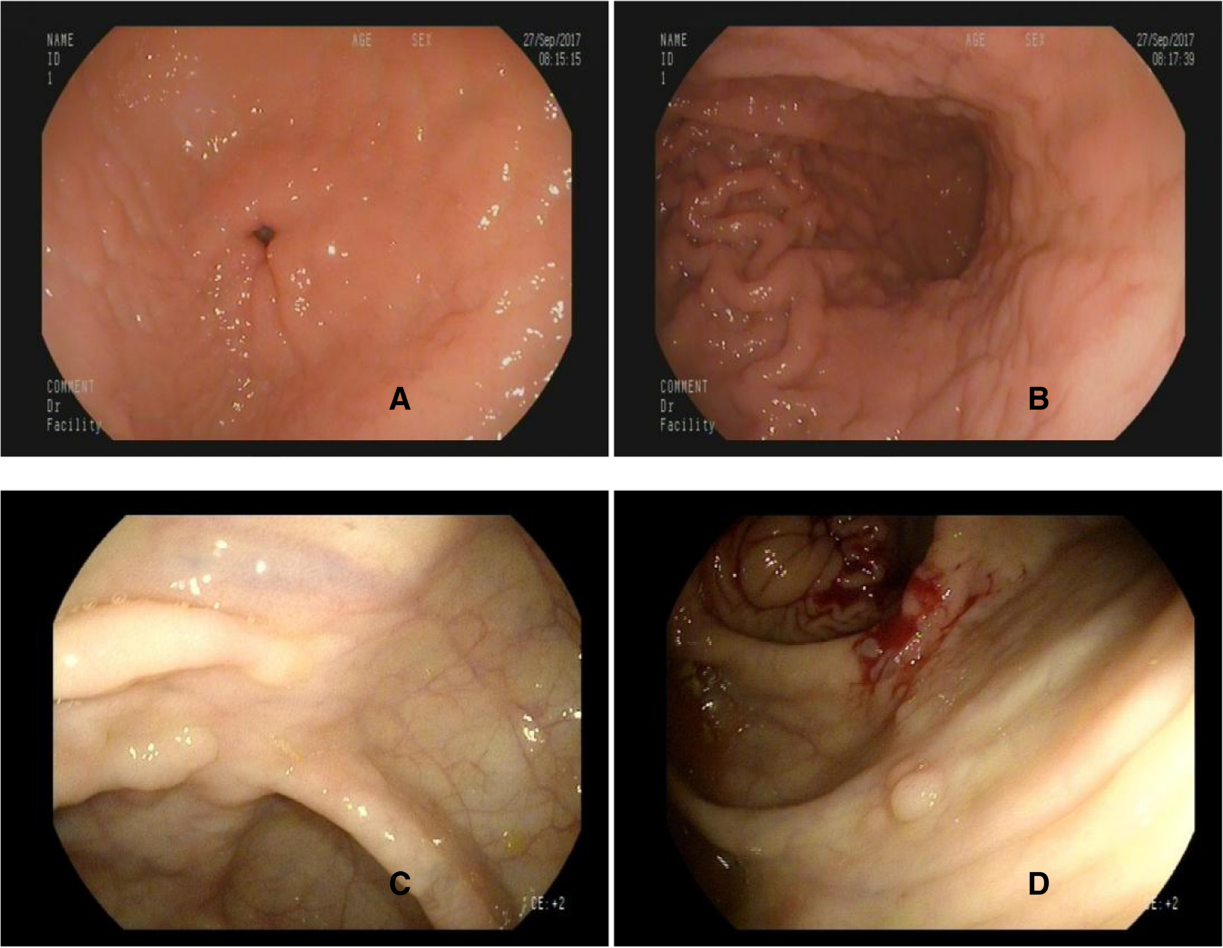
Endoscopic findings in indolent T-cell LPD of the GIT in 2017. Irregular appearance of Gastric antrum (**a**) and Gastric body (**b**) mucosa. Multiple wide pedicle polyps were found in the ileocecal (**c**) and ascending colon (**d**)

The patient was a 46-year-old man who presented with a history of abdominal distension and dyspepsia in September 2017. The complete blood picture showed white blood cell count 4.53 × 10^9^ with neutrophils 47%, lymphocytes 44%; hemoglobin, 12.3 g/dl; hematocrit36.5%; and platelet count 109 × 10^9^/ L. Flow cytometric analysis performed on peripheral blood specimens showed CD3+ cells 60.33%, CD3 + CD4+ cells 24.97%,CD3 + CD8+ cells 33.86%,NK cells 26.07%,CIK cells 18.51%,B cells 8.23%. Ultrasound images showed that the spleen is slightly enlarged with no hepatomegaly. In the year 2012, Endoscopic examinations found ileal mucosal inflammation. In 2015, endoscopic examination showed patchy erythema of the gastric fundus, and two polyps (2-3 mm) in the ascending colon and rectum. In 2017, endoscopic examination revealed rough hyperemic gastric antrum and body mucosa. Eight wide pedicle (2-3 mm) polyps were seen in the ileocecal junction and ascending colon. The sigmoid colorectal mucosa was congested and there were no ulcers or masses (Fig. 1). The patient was not given any chemotherapy and was followed up closely with no evidence of disease progression. As to date, the patient remained asymptomatic without any treatment.

### Pathological, immunophenotypic, and molecular findings

**Fig. 2:**
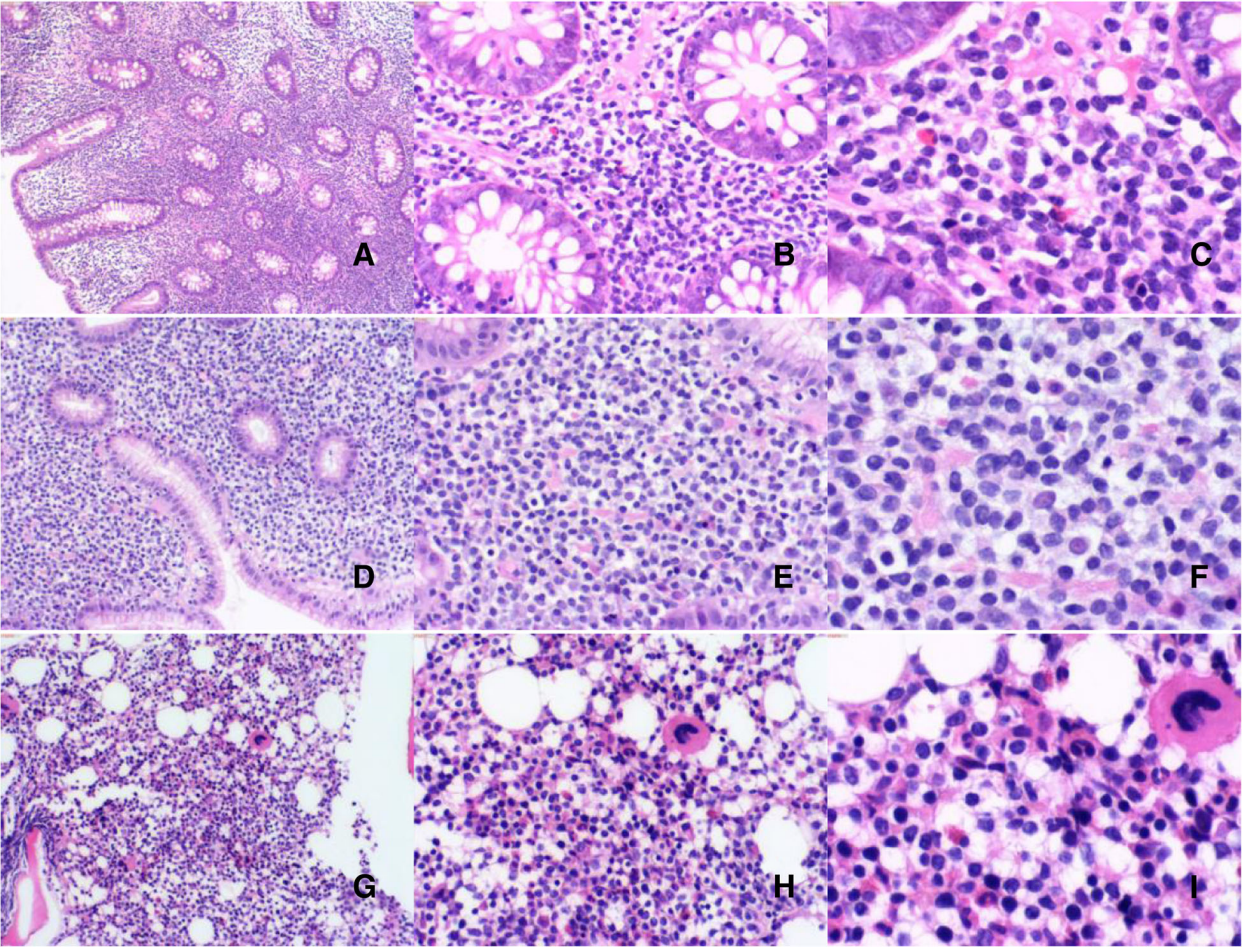
The biopsies (2017) from colon (**a,b**) and stomach (**c,d**) showed small lymphocytes with scant pale cytoplasm diffuse by infiltrating the lamina propria without cryptal destruction (**e,f**). The lymphoid cells are small and monomorphic with pale cytoplasm. Bone marrow (**g-i**) biopsy specimen showed similar patchy or interstitial infiltrate of small lymphocytes. Ileal, stomach and colon biopsies in 2015 showed similar non-destructive small lymphoid infiltrates

The following GIT biopsy and bone marrow specimens were available for review: ileum (2012), stomach (2015), colon (2015), stomach (2017), colon (2017), bone marrow (2017). All biopsy specimens showed diffuse or patchy infiltrates of predominantly small sized lymphocytes in the lamina propria with focal infiltration through the muscularis mucosae. The lymphocytes are small and monomorphic with round or angulated nucleus exhibiting fine chromatin, ambiguous nucleoli and scant to moderate pale cytoplasm (Fig. 2). The glands were displaced by the lymphoid infiltrate without being invaded or destroyed. There is no necrosis, angioinvasion or angiodestruction. Scattered plasma cells were seen in the superficial lamina propria. Mitotic activity was low in all specimens.

**Fig. 3:**
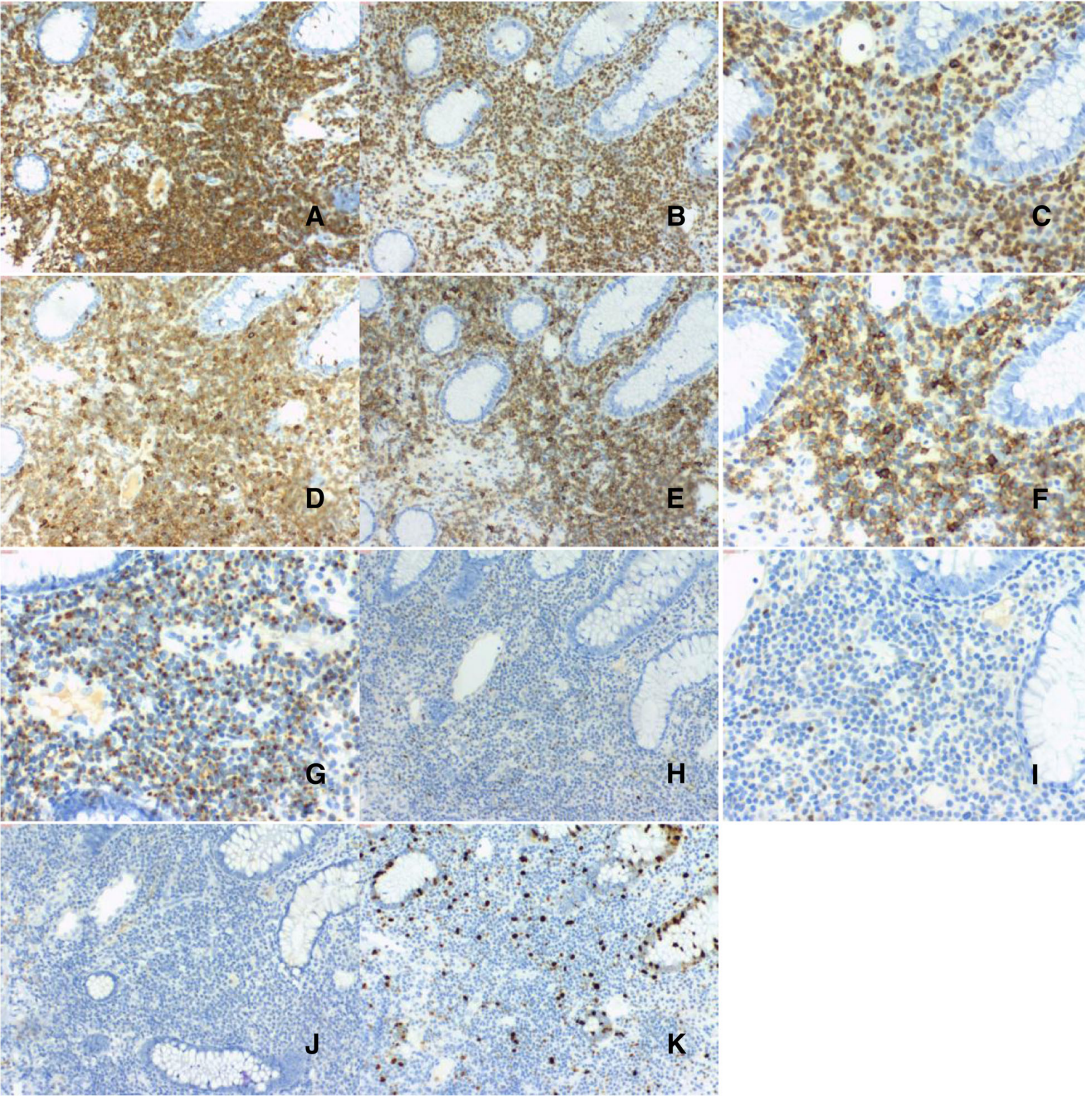
Biopsy specimen from the colon (2017). The lymphocytes express CD2 (**a**), CD3 (**b,c**), CD5(d), CD20 (**e,f**), and TIA-1 (**g**). Staining was negative for PAX5 (**h**,**i**) and CD56(J). The Ki67 (**k**) proliferative index was low

Repeated immunohistochemical staining showed that the lymphoid cells were positive for CD3, CD20, CD5, CD43, CD7, CD2, TIA1 but negative for CD4, CD8, PAX5, CD56, cyclinD1, graB, βF1. In addition, the T-cell infiltrate showed slightly dimmer expression of CD20 than the background normal B-cells. Ki-67(MIB1) proliferative index was less than 10% (Fig. 3). In situ hybridization for EBER was negative.

**Fig. 4:**
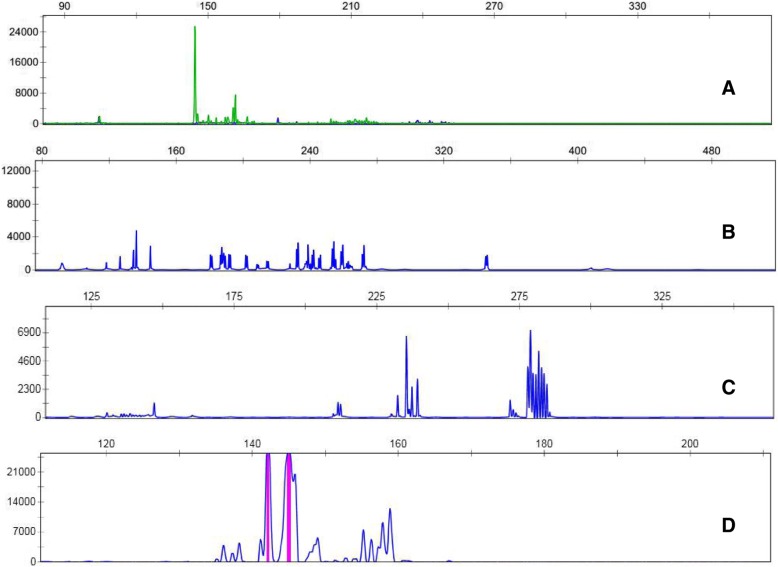
PCR analysis for TCR γ receptor and IgH gene rearrangement on formalin-fixed, paraffin-bedded tissue sections from colon biopsy specimens (2017). Clonal TCRγ receptor gene rearrangement was found (**a**), but IgH(**b**), IgK (**c**), IgL (**d**) gene rearrangement was not detected

Multiple polymerase chain reactions (PCR) for TCRß, TCRδ, TCRγ and IgH, IgK, IgL gene rearrangement were performed on biopsies from the stomach (2017) and the colon (2017). The same clonal TCRγ gene rearrangement was found though TCRß and TCRδ were germline, confirming clonal T-cell proliferation. IgH, IgK, IgL gene rearrangement was not detected (Fig. 4).

Bone marrow biopsy obtained in 2017 showed small patchy and interstitial lymphocytic infiltrates composed of similar small round lymphocytes representing less than 20% of the medullary spaces in the biopsy. Immunohistochemistry showed that the cells were positive for CD3, CD20, and negative for CD56, PAX5.

## Discussion

We present an unusual case of T-cell LPD of the GIT with aberrant expression of the B-cell marker CD20. The lesion involved multiple sites in the GIT including stomach, ileum, colon and the bone marrow. The patient had no significant past medical history and presented with persistent patches of erythema and small polyps at multiple sites in the GIT. Repeated biopsies spanning over 5 years showed involvement of the lamina propria by a non-destructive infiltrate of small lymphocytes of the same morphology. The monotonous small round mature-appearing lymphocytes had a non-activated cytotoxic immunophenotype expressing T-cell and cytotoxic cell markers. This T-cell LPD, however, unexpectedly showed an unusual aberrant expression of the B-cell marker CD20. Molecular tests demonstrated TCRγ clonal rearrangement, but not IgH rearrangement, thus confirming the T-cell lineage.

In the past couple of decades, sporadic case reports and limited series had described primary gastrointestinal T and natural killer (NK)-cell proliferations which were variably termed indolent GIT lymphoma or enteropathy [5–7]. In 2013, Perry and his colleague reported a series of 10 cases and proposed the term “indolent T-cell lymphoproliferative disease of the GIT” [8]. The 2016 revised WHO classification added this as a provisional entity [9]. Most of the reported cases occurred in adults with the patients’ age ranging from 15 to 77 years old. Clinical manifestations included abdominal pain, diarrhea, dyspepsia, vomiting. Most cases involved the small intestine or colon with bone marrow and peripheral blood usually not being involved [9, 10]. Our case involved multiple sites of the gastrointestinal tract including stomach, small intestine and colon, with also bone marrow involvement. Despite the bone marrow involvement, the disease has not progressed or showed large cell transformation. The patient had remained well since the time of the first biopsy in 2012 without institution of chemotherapy. The unusual involvement of the bone marrow in our patient did not affect the indolent course of the disease, though further clinical follow up is required for confirmation. All reported patients showed an indolent clinical course. Some patients received chemotherapy due to misdiagnosis of enteropathy-associated intestinal T-cell lymphoma. Some other patients received anti-inflammatory agents for treatment as inflammatory bowel disease. Both types of treatment did not lead to any clinical response. Non-response to chemotherapy may partly be due to the low proliferative activity of the tumor cells.

According to immunophenotypic features, indolent T-cell lymphoproliferative disease can be subdivided into CD4+, CD8+ or CD4-CD8-. Most cases are CD8+ with many being CD4+ [9]. The double negative phenotype CD4-CD8- is the least common. Double-negativity for CD4 and CD8 is associated with a more aggressive clinical course [10]. Our case also aberrantly expressed the B-cell marker CD20 which has hitherto not been reported in indolent T-cell LPD of the GIT.

It is important to differentiate indolent T-cell LPD of the GIT from more aggressive lymphomas, either primary or those secondarily involving the GIT, as well as from inflammatory bowel diseases. Monomorphic epitheliotropic intestinal T-cell lymphoma and enteropathy-associated T-cell lymphoma show infiltration by atypical, intermediate or large-sized lymphocytes intraepithelial and transmural with a high proliferative index. The clinical presentation is generally more acute because of intestinal obstruction or perforation [11, 12]. In contrast, the lesions of indolent LPD are usually superficial without mass or destructive growth pattern, possess bland cytology and always low proliferative index. The absence of angiocentricity or angiodestruction and negative EBER can easily exclude nasal type aggressive extranodal NK/T-cell lymphoma. A few cases of indolent LPD of the GIT have a history of inflammatory bowel disease (IBD), but the relationship between IBD and indolent T-cell LPD of the GIT is still unclear at present [13, 14]. Careful histopathologic and immunophenotypic evaluation, in addition the presence of a clonal TCR rearrangement can distinguish indolent T-cell LPD from IBD [15].

Low grade B-cell lymphomas such as extranodal marginal zone lymphoma of mucosa-associated lymphoid tissue (MALT lymphoma) and mantle cell lymphoma (MCL) could also be confused with indolent T-cell LPD. MCL is particularly problematic; as MCL can present as multiple intestinal polyposis. This is particularly important as the present report shows possibility of aberrant CD20 positivity in indolent T-cell LPD. MALToma is distinguished by the presence of lymphoepithelial lesions, monocytoid lymphoid cells and distinctive immunophenotype including expression of the marginal zone marker IRTAI. MCL is distinguished by nodular, diffuse or mantle zone pattern with its special immunophenotype including cyclin D1 and S0XII positivity [9]. Though CD20 may be positive in T-cell LPD, the lack of other B-cell makers, the expression of T-cell markers and clonal T-cell receptor management distinguishes T-cell LPD from low grade B-cell lymphomas.

In summary, we report an unusual case of CD4-CD8- double negative indolent T-cell LPD of the GIT with aberrant CD20 expression and TCR γ rearrangement. CD20 positive has been found in several types of T-cell lymphomas including peripheral T-cell lymphoma, mycosis fungoides and enteropathy-type T-cell lymphoma [16]. Our case is the first report of indolent T-cell LPD of the GIT with CD20 aberrant expression. Despite persistent involvement of multiple sites of the GIT and bone marrow, our patient has not shown disease progression or high grade transformation even without any treatment.

## Conclusion

We present an unusual case of indolent T-cell lymphoproliferative disorder with aberrant CD20 expression, Recognition of this unusual immunophenotype of indolent T-cell LPD of GI helps to eschew misdiagnosis of B-cell and other high grade lymphomas and inappropriate aggressive treatment.

## Abbreviations

CIK: Cytokine-induced killer
EBER: Epstein Barr virus-encoded RNA
GIT: Gastrointestinal tract
GraB: Granzyme
IBD: Inflammatory bowel disease
IgH: Immunoglobulin heavy chain
LPD: Lymphoproliferative disorder
MALT: Mucosa-associated lymphoid tissue
MCL: Mantle cell lymphoma
NK: Natural killer
PCR: Polymerase chain reactions
TCR γ: T-cell receptor γ
